# Clinical practice of cardiovascular magnetic resonance: position statement of the Society for Cardiovascular Magnetic Resonance

**DOI:** 10.1186/s12968-019-0592-x

**Published:** 2019-12-23

**Authors:** The SCMR Board of Trustees

**Affiliations:** Society for Cardiovascular Magnetic Resonance (SCMR), 19 Mantua Rd, Mt. Royal, NJ 08061 USA

The Society for Cardiovascular Magnetic Resonance (SCMR) is the international professional society that strives to improve cardiovascular health by advancing the field of cardiovascular magnetic resonance (CMR). Through education, research, and advocacy, the Society promotes the highest standards of quality in CMR. As such, the Society works to ensure that CMR professionals are educated and well-qualified to safely perform and accurately interpret CMR studies for our patients. Professional competency is key to our mission and is fostered in every activity promoted by the Society.

The Society is the home for all CMR professionals (clinicians, scientists, engineers, technologists, nurses, administrators, and trainees) including those involved with CMR sequence development, basic and clinical research, procedural planning, image acquisition and analysis, interpretation, and the delivery of a clinical service. All these professionals are equally valued and supported by our Society, and we champion those who work collaboratively with a patient-centered approach. The Society recognizes that CMR is part of both the radiology and cardiology curricula, and that the appropriate interpretation of CMR studies requires training in aspects of both radiology and cardiology. By statute, the board of the Society has a balanced representation of cardiologists and radiologists aimed at supporting both in the clinical practice of CMR.

The Society aims to advance the field by qualifying and empowering suitably trained physicians to deliver on the promise of CMR to improve cardiovascular health across practice settings. The Society has published guidelines for training in CMR, which complement international training curricula in both radiology and cardiology. The Society supports laboratory accreditation and encourages imaging physicians, regardless of specialty, to earn CMR certification. The Society encourages training in multidisciplinary competencies for radiologists, cardiologists, and other imaging physicians wishing to practice clinical CMR.

Varied practice models for delivering clinical CMR services exist around the world led by radiologists, cardiologists, or jointly, in the public and private sectors. The Society supports all of these organizational models and it objects to practices that place barriers to CMR participation based solely on specialty. The Society strongly believes that clinical safety and optimal patient care can be best achieved when appropriately trained and qualified physicians provide CMR imaging services regardless of their specialty.

## Data Availability

Not applicable.

## Abbreviations

CMR: Cardiovascular magnetic resonance
SCMR: Society for Cardiovascular Magnetic Resonance

